# Small diameter blood vessels with controllable micropore structure induced by centrifugal force for improved endothelialization

**DOI:** 10.1002/elsc.201900123

**Published:** 2020-01-21

**Authors:** Jinge Li, Qinwei Gao, Zhaobin Chen, Xiaoniu Yang

**Affiliations:** ^1^ Polymer Composites Engineering Laboratory Changchun Institute of Applied Chemistry Chinese Academy of Sciences Changchun Jilin P. R. China; ^2^ State Key Laboratory of Polymer Physics and Chemistry Changchun Institute of Applied Chemistry Chinese Academy of Sciences Changchun Jilin P. R. China; ^3^ School of Applied Chemistry and Engineering University of Science and Technology of China Hefei Anhui P. R. China

**Keywords:** centrifugal force, endothelialization, micropore structure, polyurethane, small diameter blood vessel

## Abstract

The micropore structure is prerequisite for fast and durable endothelialization of artificial small diameter blood vessels (ASDBVs). Although some methods, such as salt leaching, coagulation, and electrospinning, have been developed to construct micropores for ASDBVs, the uncontrollability of the structure and the complicated procedures of the process are still the issues to be concerned about. In this study, a compact device based on the principle of centrifugal force is established and used to prepare polyurethane (PU) ASDBVs with micropore structures by blasting different porogens. It is found that the glass beads could construct micropores with regular round shape, uniform distribution, and controllable size (60–350 µm), which significantly improves the endothelialization of PU‐based ASDBVs, especially when the pore size is about 60 µm. This method is easy‐accessible and wide‐applicable, which provides a new pathway for the research and development of ASDBVs.

**Abbreviations**
ASDBV: artificial small diameter blood vessels
ECs: endothelial cells
Eptfe: polytetrafluoroethylene
PCL: polycaprolactone
PCU: polycarbonate urethane
PET: polyethylene terephthalate
PU: polyurethance

## 1 INTRODUCTION

Although coronary artery bypass graft (CABG) is so far the prior choice for patients with severe coronary artery diseases, its applications are restricted due to the limited source of autologous vessels 1, which promotes the research and development of artificial small diameter blood vessels (ASDBVs, inner diameter < 6 mm). Currently, the ASDBVs made of synthetic polymers, such as polyurethane (PU), expanded polytetrafluoroethylene (ePTFE), polyethylene terephthalate (PET), polycaprolactone (PCL), and polyglycolic acid (PGA), are the most used alternatives to natural vessels in CABG, but the poor blood patency rate caused by thrombosis after implantation still needs to be investigated 2.

To date, miscellaneous surface modifications have been developed to provide ASDBVs with anti‐thrombosis function, such as changing the hydrophilicity and electronegativity, grafting bioactive molecules, and promoting the endothelialization process 3. Endothelium is the confluent layer of functional endothelial cells (ECs) formed on the luminal surface of blood vessels, which locates between the vessel's tissue and blood and plays a very important role in regulating the anti‐thrombotic function of natural blood vessels 4. It is currently well recognized that the endothelialization is the most effective and powerful route to realize the anti‐thrombosis of ASDBVs 3, 5. Micropore structure has long been reported to be the prerequisite for the ASDBVs because it can anchor ECs and is beneficial for the rapid and durable endothelialization 6, 7. For example, more rapid endothelialization rate was demonstrated by 60 µm of pores size rather than 30 µm for ePTFE vascular prostheses 8. Similarly, 60 and 90 µm of pore size were superior to 20 µm and 40 µm for porous ePTFE grafts in terms of neoendothelial healing 9. Obviously, appropriate size of micropores is needed for better endothelialization of ASDBVs. The micropore structure of ASDBVs could be realized by some methods, for example, salt leaching 10, coagulation 11, electrospinning 12, and phase‐inversion 13. Although these techniques could construct the micropores, the uncontrollability of the structure (for the former two) and the complicated procedures (for the latter two) always trouble the researchers in the field.

In this study, a simple and cost‐effective method for the fabrication of ASDBVs with micropore structures is proposed, which is based on the principle of centrifugal force and realized on a self‐established device. Through adjusting the operation parameters and porogen type, uniform and controllable micropore is created for PU‐based ASDBVs, which is beneficial for endothelialization process, and the obtained microporous ASDBVs show the biomechanical strength and compliance better than or similar to that of saphenous vein. This method is applicable for the polymers that are soluble in organic solvents.

**PRACTICAL APPLICATION**

A method based on the principle of centrifugal force and sand blasting is proposed, and a compact device is built up in the laboratory to fabricate microporous artificial small diameter blood vessels (ASDBVs). Through adjusting the operation conditions and screening porogens, micropores with regular round shape, uniform distribution, and controllable size (60–350 µm) could be realized, and the endothelialization is significantly improved. This method is applicable not only for the polyurethane‐based ASDBVs, but also for the solvent‐soluble polymeric materials. This method is simple, cost‐effective, easy‐accessible, and wide‐applicable, which provides the researchers with a new pathway to prepare ASDBVs in the research and development.

## 2 MATERIALS AND METHODS

### 2.1 Materials

Polycarbonate urethane (PCU, DSM, the Netherlands), polycaprolactone (PCL, Sigma–Aldrich, China), and polyethylene terephthalate (PET, DuPont, USA) were purchased and used without further purification. NaCl particles were obtained by grinding NaCl crystals and collecting in the range of 150–300 µm; paraffin spheres were prepared according to Grenier's method 14, and collected in the range of 150–300 µm; glass beads (63–75 µm, 90–106 µm, 150–180 µm, and 250–355 µm) were purchased from Fuji Manufacturing Co., Ltd., Japan. Other chemicals were used directly as received unless otherwise stated.

### 2.2 Preparation of ASDBVs

**Figure 1:**
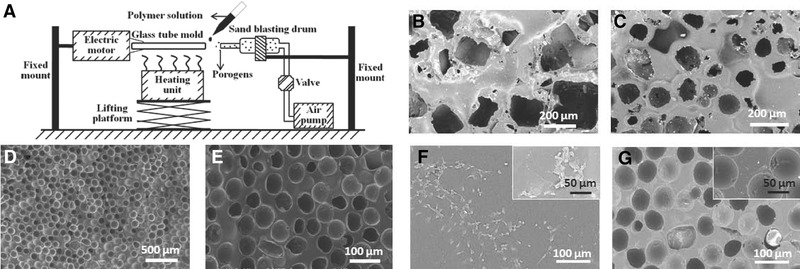
(A) Diagram of the device for the preparation of microporous ASDBVs. (B), (C), and (D) and (E) are the micropore structures of the PCU‐based ASDBVs prepared by NaCl particles, paraffin spheres, and glass beads, respectively. (F) and (G) are the endothelialization behaviors of ECs on the smooth (PCU0) and microporous PCU‐based ASDBVs with pore size of 63–75 µm (PCU1) after 48 h incubation, respectively

In order to fabricate the microporous ASDBVs, a compact device was first built up in the laboratory, which mainly consisted of electric motor, heating unit, and sand blasting drum, as shown in Figure 1A 15, 16. The electric motor could drive the glass tube mold to rotate at different speed, and the direction of the rotating motor was manually adjusted up and down (Figures S1 and S2) so that the polymer solution and porogens could be evenly distributed in both axial and circumferential directions. The heating unit was used to remove the organic solvents in the polymer solution, and the sand blasting drum carried and fed the porogens into the mold.

The polymers were dissolved in organic solvents (PCU, PCL, and PCU/PCL with 1:1 of mass ratio in *N,N*‐dimethylformamide (DMF), PET in trifluoroacetic acid (TFA) to form solutions with 10% concentration). The polymer solution was dropwise added into the glass tube mold with a certain rotation speed (typically 1000 rpm) at 60°C, which will uniformly cover the inner surface of the glass tube under the traction of centrifugal force by adjusting the angle of the horizontal direction of the tube mold. After the solvent in the film was evaporated to some extent (semi‐dried), the porogens, such as NaCl particles and glass beads, were blasted into the tube mold. The blasting speed or gas flow rate within the range of 3–10 mL/s could effectively blow the porogens into the mold and make them to adhere to the surface of the polymer film. It is worth mentioning that the gas flow rate should be properly adjusted according to the type and size of porogens in the preparation of microporous ASDBVs (Table S1). Then, raising the temperature to 90°C and keeping the rotation speed constant to completely evaporate the solvent. As an exception, the operation for paraffin spheres proceeded under room temperature due to their low melting point. The ASDBVs were gently removed from the mold and then ultrasonically treated three times for 30 min each to remove the porogens (NaCl particles and glass beads in water, paraffin spheres in hexane). The obtained microporous ASDBVs were dried at 50°C in oven for 24 h.

### 2.3 Characterization

The morphologies of the inner surfaces of the microporous ASDBVs were observed by field emission scanning electron microscopy (FE‐SEM, XL 30, FEI Company) with an accelerating voltage of 10 kV. The cross sections were characterized by digital microscope (VHX, Keyence).

The tensile strength, burst pressure, and dynamic compliance of ASDBVs were tested and calculated according to ISO 7198:2017.

The endothelialization behaviors of ASDBVs were assessed by human umbilical vein endothelial cells (HUVECs). The prepared ASDBVs were cut into disks with a diameter of 15 mm, which were sterilized and flattened by pressing PTFE ring (inner diameter > 10 mm) on the top microporous surface in the 24‐well tissue culture plate. The cells were seeded at a density of 3 × 10^4^ cells per well and incubated at 37°C for 48 h, after which the numbers of the cells were measured by cell counting kit‐8 (CCK‐8). The cytotoxicity of ASDBVs was tested in vitro by NIH 3T3 with an extraction method according to modified ISO 10993–5: 2009 17. The extracts concentration was 0.2, 1.0, and 2.0 cm^2^/mL, respectively, and the normal medium was used as the negative control (regarded as 100%).

## 3 RESULTS AND DISCUSSION

In this study, PCU is selected as the matrix to prepare microporous ASDBVs due to its easy processing, excellent mechanical strength, and good biostability and biocompatibility 18. Figure 1B, C, and D and E are the SEM pictures of the inner surfaces of ASDBVs prepared by NaCl particles, paraffin spheres, and glass beads, respectively. It can be seen that all the porogens used in this study could construct micropore structure on PCU surfaces, but NaCl particles and paraffin spheres are not good choice because the micropores they produced are shape‐irregular, distribution‐uneven, and size‐uncontrollable. In contrast, the micropores obtained by glass beads show regular round shape, even distribution, and uniform size (63–75 µm, Figure 1D), and smooth boundaries at higher magnification (Figure 1E). Actually, same topography of the surface could also be observed for other PCU‐based ASDBVs prepared by using glass beads in the range of 90–355 µm (shown in Table 1 and Figure S3).

**Table 1:** Fundamental properties of PCU‐based ASDBVs

Performance	PCU0	PCU1	PCU2	PCU3	PCU4
ID (mm)	4.79 ± 0.01	4.62 ± 0.02	4.67 ± 0.03	4.40 ± 0.10	4.54 ± 0.05
Wall thickness (mm)	0.11 ± 0.01	0.19 ± 0.01	0.17 ± 0.02	0.30 ± 0.05	0.23 ± 0.03
Pore size (µm)	0	63–75	90–106	150–180	250–355
Proliferation rate of ECs (%)	100.00 ± 1.01	291.04 ± 0.59	302.83 ± 1.26	144.34 ± 1.59	116.51 ± 1.95
Tensile strength (MPa)	39.64 ± 6.83	23.83 ± 3.48	14.15 ± 1.10	9.94 ± 2.72	8.06 ± 1.00
Burst pressure (kPa)	271.9 ± 21.4	210.5 ± 14.2	190.8 ± 21.3	145.1 ± 9.7	108.4 ± 18.6
Compliance (%/100 mmHg)	3.42 ± 0.30	4.65 ± 0.28	7.10 ± 0.88	9.40 ± 1.24	11.31 ± 2.16
Cytotoxicity (2.0 cm^2^/mL, %)	77.95 ± 1.30	80.47 ± 1.44	77.89 ± 1.24	80.99 ± 1.27	81.39 ± 0.39

The endothelialization behaviors of the prepared microporous ASDBVs are investigated in the medium of HUVECs. It is found that in comparison to the smooth sample, all the microporous ASDBVs show higher proliferation rate of ECs after 48 h incubation (Table 1). In other words, the micropore structure is beneficial for the EC proliferation, but the extent of which largely depends on the pore size: the optimum proliferation rate is reached at 60–100 µm (PCU1 and PCU2), which is in good agreement with the literature reports (60–90 µm) 8, 9 and three times higher than the control PCU0, while overlarge pore results in lower increases (PCU3 and PCU4). The proliferation of ECs on PCU0 and PCU1 is observed by SEM (Figure 1F and G), which shows that the ECs on the surface of PCU1 distribute not only massively but also uniformly in comparison to that of PCU0, demonstrating the promoted endothelialization of the micropore structure.

The microporation process has also influence on other properties of PCU‐based ASDBVs, as shown in Table 1. It is reasonable that as the pore size increases, the tensile strength and the burst pressure decrease, while the compliance increases. It is found that for PCU1 with 63–75 µm of micropore size and best endothelialization, 4.65 ± 0.28% of compliance is similar to that of saphenous vein (4.4%), 23.83 ± 3.48 MPa of tensile strength, and 210.5 ± 14.2 kPa (equivalent of ca. 1580 mm Hg) of burst pressure are also within the safe range 18. The micropore structure does not impact on the cytotoxicity of the ASDBVs, approximate or over 80% of NIH 3T3 cells are recovered for all the samples under different conditions (Table 1 and Figure S4), which is at the same level of the smooth sample PCU0.

**Figure 2:**
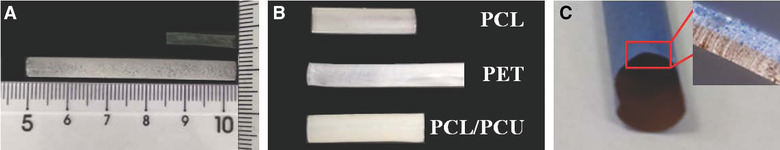
(A) PCU‐based ASDBVs with different length and inner diameter. (B) PCL, PET, and PCL/PCU blend ASDBVs prepared by this method. (C) PCU‐based ASDBV with bilayer wall structure, the thicknesses of inner and outer layers are 47 µm, respectively

This method for fabricating ASDVBs with micropore structure is based on centrifugal force and sand blasting, which is simple, cost‐effective, and easy accessible to the lab researchers. Through adjusting the type and size of the porogens, different shape and size of micropore structure could be realized, and the tunable depth of the micropore could also be reached by changing centrifugal force (Figure S5 and Table S2). The micropore depth increases with the increase in centrifugal force, meaning the increased specific surface area of inner layer of ASDBVs, which is conductive to ECs proliferation. Currently, a series of PCU‐based ASDBVs (length < 50 mm, inner diameter 3–6 mm) have been prepared in the authors’ laboratory (Figure 2A). Actually, this method is applicable for all the thermoplastic polymers that are soluble in organic solvents such as PCL, PET, and polymer blends (PCL/PCU; Figure 2B). In addition, the ASDBVs with mimic multiplayer structure to the natural vessels could also be fabricated as shown in Figure 2C. It is worth mentioning that ASDBVs prepared with centrifugal force could be endowed with biological functions, such as anti‐thrombus and anti‐inflammation, through the selection of functional materials or embedding drugs. For example, a kind of selenium‐containing polyurethane 17 could be used to prepare inner layer of ASDBVs by this centrifugal force method. Thus, this ASDBV would continue to release nitric oxide to inhibit platelet adhesion and activation. Moreover, anticoagulants, including heparin and aspirin, are allowed to be embedded in the wall of ASDBVs prepared by this centrifugal method to achieve the long‐term anticoagulant effect through the slow release of drugs. Of course, work on these features is ongoing, and the results will be shared with researchers in the near future. To sum up, in view of the accessibility, simplicity, and effectiveness, it is believed that the method reported here could provide the researchers with a new solution for ASDBVs studies.

## 4 CONCLUDING REMARKS

The micropore structure in the wall of ASDBVs is considered to be the premise of endothelialization 6, 7, which is recognized as one of the most effective methods of long‐term anti‐thrombosis of vascular substitutes 3, 5. At present, the simple and rapid method to achieve endothelialization of ASDBVs has been the focus of many researchers 19, 20.

In this study, a new method based on centrifugal force and sand blasting is proposed and a compact device is built up for the fabrication of microporous ASDBVs. Through adjusting the operation conditions and screening porogens, micropores with regular round shape, uniform distribution, and controllable size (60–350 µm) could be realized on PCU‐based ASDBVs, which significantly improve the endothelialization. This method is simple, cost‐effective, easy‐accessible, and wide‐applicable, which would provide a new pathway for the research and development of ASDBVs.

## CONFLICT OF INTEREST

The authors have declared no conflict of interest.

## Supporting information

supporting information
