# Supplementary material for: Small diameter blood vessels with controllable micropore structure induced by centrifugal force for improved endothelialization
Source: Eng Life Sci. 2020 Jan 21;20(5-6):181–5. doi: 10.1002/elsc.201900123 (PMC7447902; doi:10.1002/elsc.201900123)
Supplement: Supplementary file 1 — supporting information [file ELSC-20-181-s001.pdf]

Supporting Information

Small diameter blood vessels with controllable micropore structure induced by centrifugal force for improved endothelialization

Jinge Li[1]

Qinwei Gao[2,3]

Zhaobin Chen[1]

Xiaoniu Yang[1,2]

[1]Polymer Composites Engineering Laboratory, Changchun Institute of Applied Chemistry, Chinese Academy of Sciences, Changchun, Jilin 130022, P. R. China

[2]State Key Laboratory of Polymer Physics and Chemistry, Changchun Institute of Applied Chemistry, Chinese Academy of Sciences, Changchun, Jilin 130022, P. R. China

[3]School of Applied Chemistry and Engineering, University of Science and Technology of China, Hefei, Anhui 230026, P. R. China

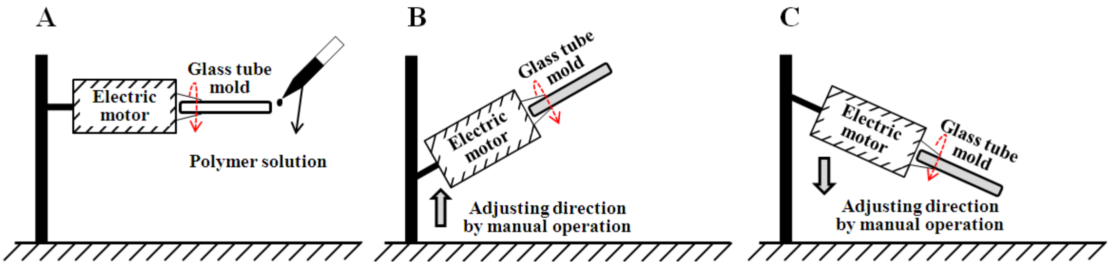

Figure S1. The side of the driving unit (glass tube mold) could be manually adjusted up and down to make the even distribution of the polymer solution in the mold.

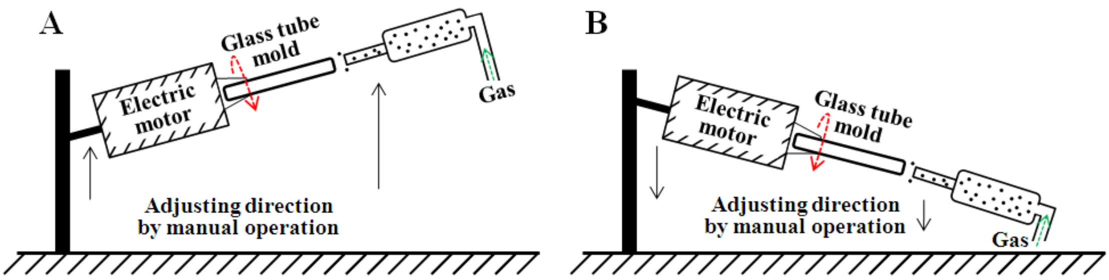

Figure S2. The side of the driving unit (glass tube mold) could be manually adjusted up and down to make the even distribution of the porogens in the mold.

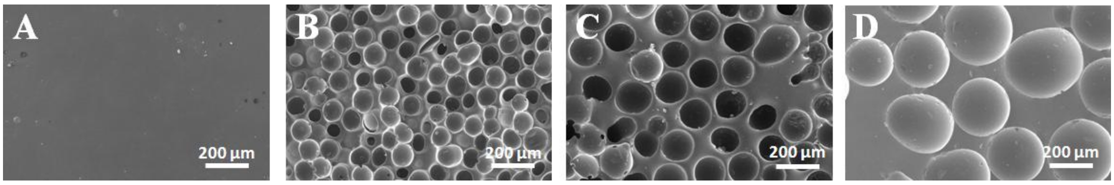

Figure S3. The SEM pictures of inner surfaces of smooth (A) and microporous PCU-based ASDBVs with pore sizes of 90-106 μm (B), 150-180 μm (C), and 250-355 μm (D).

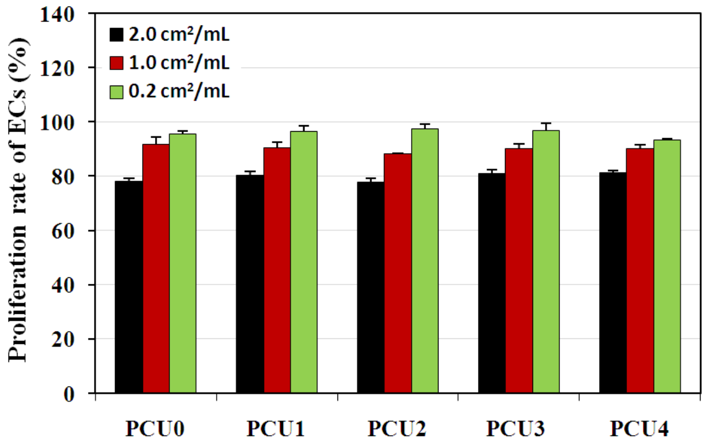

Figure S4. The cytotoxicity of the PCU-based ASDBVs.

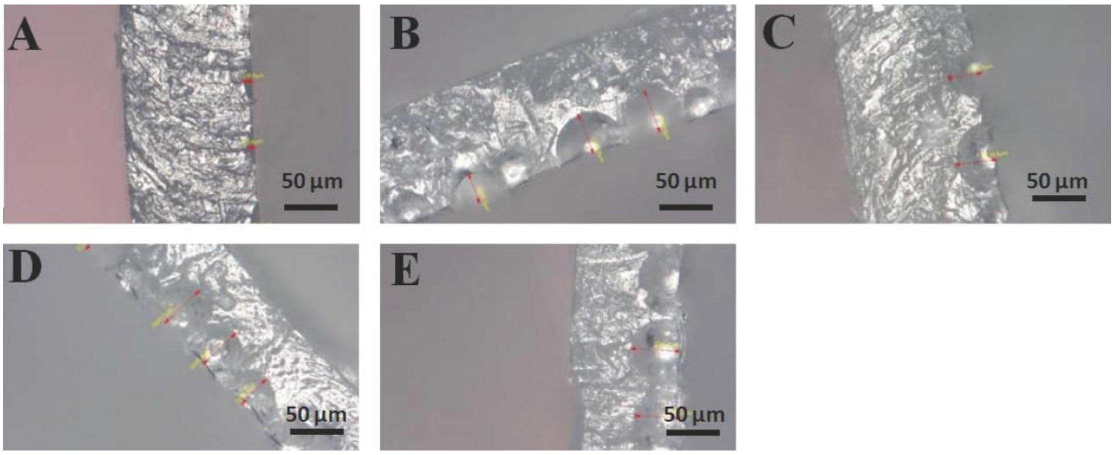

Figure S5. The optical microscope pictures of the cross sections of microporous PCU1 ASDBV prepared with 1.12×10^{-3} g (A), 0.70 g (B), 2.80 g (C), 6.29 g (D), and 11.20 g (E) of centrifugal forces (g ≈ 9.8 m s^{-2}), respectively.

Table S1. The gas flow rate used for porogens of different types and sizes

Porogens	Glass beads				NaCl particles	Paraffin spheres
Size (μm)	63-75	90-106	150-180	250-355	150-300	150-300
Gas flow rate (mL/s)	5.0	5.0	6.5	7.0	6.0	3.0

Gas flow rate or blasting speed within the range of 3-10 mL/s could effectively blow the porogens, including glass beads, NaCl particles, and paraffin spheres, into the mold and make them to adhere to the surface of the polymer film. If the flow rate was too low (<2mL/s), the porogens could not be continuously injected into the mold; if the flow rate was too high (>15mL/s), the gas would destroy the flatness of the polymer film in the mold. It is worth mentioning that the gas flow rate should be properly adjusted according to the type and size of porogens in the preparation of microporous ASDBVs.

Table S2. The depth of micropores of PCU1 ASDBV measured from Figure S5

	PCU1				
Motor speed (rpm)	20	500	1000	1500	2000
Centrifugal force (g)	1.12×10^{-3}	0.70	2.80	6.29	11.20
Pore depth (μm)	8.1 ±3.2	30.3 ±6.9	35.9 ±4.7	41.3 ±2.1	45.3 ±2.6
